# Supplementary material for: Mother-to-child transmission of HIV infection and its associated factors in the district of Bilene, Gaza Province—Mozambique
Source: PLoS One. 2021 Dec 10;16(12):e0260941. doi: 10.1371/journal.pone.0260941 (PMC8664209; doi:10.1371/journal.pone.0260941)
Supplement: S1 File — (PDF) [file pone.0260941.s001.pdf]

Health Facility	Residence	Ocupation	education level	Age (years)	Number of deliveries	Date of 1st antenatal visit (ANC)	Gestational age (inweeks) at the opening o	Total of ANC visits	Date of ART initiation	Adherence to ART	WHO clinical stage	Nutritional status during gestation/breastfeeding
CENTRO DE SAUDE DA PRAIA	Chilengue	Domestic worker	primary level	26	3	2nd trimester	24	≥4	2nd gestational trimester	Adherent	1	Normal
CENTRO DE SAUDE DA PRAIA	Mahungo	Domestic worker	Basic secondary level	22	2	3rd trimester	28	≥4	Before gestation	Adherent	1	Normal
CENTRO DE SAUDE DA PRAIA	No information	No information	No information	25	2	2nd trimester	16	≥4	2nd gestational trimester	Defaulter	3	Normal
CENTRO DE SAUDE DA PRAIA	Tsoveca	Domestic worker	No formal education	40	≥4	3rd trimester	28	≥4	3rd gestational trimester	Defaulter	1	Normal
CENTRO DE SAUDE DA PRAIA	No information	No information	No information	20	2	2nd trimester	20	≥4	2nd gestational trimester	Lost to follow up	1	Normal
CENTRO DE SAUDE DA PRAIA	Chimembanine	Domestic worker	Basic secondary level	20	1	2nd trimester	19	≥4	Before gestation	Adherent	3	Normal
CENTRO DE SAUDE DA PRAIA	Nhuane	Self employed	primary level	29	3	2nd trimester	16	≥4	Before gestation	Adherent	1	Normal
CENTRO DE SAUDE DA PRAIA	Tsoveca	Domestic worker	primary level	30	3	2nd trimester	18	≥4	2nd gestational trimester	Adherent	1	Normal
CENTRO DE SAUDE DA PRAIA	Tsoveca	Domestic worker	Basic secondary level	30	≥4	2nd trimester	13	≥4	Before gestation	Adherent	1	Normal
CENTRO DE SAUDE DA PRAIA	Tsoveca	Domestic worker	No formal education	42	≥4	3rd trimester	28	2	Before gestation	Defaulter	1	Normal
CENTRO DE SAUDE DA PRAIA	Mahungo	Domestic worker	Basic secondary level	23	≥4	2nd trimester	14	≥4	2nd gestational trimester	Adherent	1	Normal
CENTRO DE SAUDE DA PRAIA	Mahungo	Student	primary level	25	≥4	2nd trimester	20	≥4	Before gestation	Adherent	1	Normal
CENTRO DE SAUDE DA PRAIA	Nhabanga	Self employed	Basic secondary level	28	≥4	2nd trimester	20	2	2nd gestational trimester	Defaulter	1	Normal
CENTRO DE SAUDE DA PRAIA	Chilengue	Student	Medium secondary level	24	1	3rd trimester	25	≥4	Before gestation	Adherent	1	Normal
CENTRO DE SAUDE DA PRAIA	Mahungo	Domestic worker	primary level	26	2	2nd trimester	22	≥4	Before gestation	Adherent	1	Normal
CENTRO DE SAUDE DA PRAIA	Chilengue	Domestic worker	Basic secondary level	30	2	2nd trimester	16	≥4	2nd gestational trimester	Adherent	1	Normal
CENTRO DE SAUDE DA PRAIA	Mivane	Self employed	primary level	28	1	2nd trimester	23	≥4	2nd gestational trimester	Adherent	1	Normal
CENTRO DE SAUDE DA PRAIA	Chihacho	Domestic worker	primary level	29	3	2nd trimester	16	≥4	Before gestation	Adherent	2	Normal
CENTRO DE SAUDE DA PRAIA	Nhabanga	Domestic worker	primary level	38	3	1st trimester	12	≥4	Before gestation	Adherent	1	Normal
CENTRO DE SAUDE DA PRAIA	Chilengue	Domestic worker	Basic secondary level	24	2	1st trimester	12	≥4	Before gestation	Adherent	1	Normal
CENTRO DE SAUDE DA PRAIA	Chilengue	Domestic worker	primary level	28	1	1st trimester	12	≥4	Before gestation	Adherent	1	Normal
CENTRO DE SAUDE DA PRAIA	Chilengue	Domestic worker	No formal education	42	≥4	3rd trimester	28	2	Before gestation	Adherent	1	Normal
CENTRO DE SAUDE DA MACIA	Bairro 4 Macia	Domestic worker	primary level	36	≥4	2nd trimester	17	≥4	2nd gestational trimester	Adherent	2	Normal
CENTRO DE SAUDE DA MACIA	Bairro 1 Macia	Domestic worker	Medium secondary level	28	2	2nd trimester	20	≥4	2nd gestational trimester	Adherent	2	Normal
CENTRO DE SAUDE DA MACIA	Chimonzo	public servant	Medium secondary level	33	3	2nd trimester	17	≥4	2nd gestational trimester	Adherent	2	Normal
CENTRO DE SAUDE DA MACIA	Incoluane	Domestic worker	primary level	24	2	3rd trimester	27	≥4	Before gestation	Adherent	1	Normal
CENTRO DE SAUDE DA MACIA	Bairro 6 Macia	Domestic worker	Basic secondary level	21	2	2nd trimester	21	≥4	2nd gestational trimester	Adherent	1	Normal
CENTRO DE SAUDE DA MACIA	Bairro 3 Macia	Domestic worker	primary level	27	1	1st trimester	12	≥4	1st gestational trimester	Adherent	1	Normal
CENTRO DE SAUDE DA MACIA	Bairro 1 Macia	Domestic worker	No formal education	27	1	2nd trimester	20	≥4	Before gestation	Adherent	2	Normal
CENTRO DE SAUDE DA MACIA	Bairro 2 Macia	public servant	Medium secondary level	27	2	3rd trimester	25	≥4	3rd gestational trimester	Adherent	2	Normal
CENTRO DE SAUDE DA MACIA	Bairro 5 Macia	Domestic worker	primary level	30	3	2nd trimester	24	≥4	2nd gestational trimester	Adherent	1	Normal
CENTRO DE SAUDE DA MACIA	Bairro 5 Macia	Domestic worker	No formal education	29	≥4	2nd trimester	24	≥4	Before gestation	Adherent	2	Normal
CENTRO DE SAUDE DA MACIA	Bairro 2 Macia	Domestic worker	Medium secondary level	27	1	2nd trimester	16	≥4	2nd gestational trimester	Adherent	1	Normal
CENTRO DE SAUDE DA MACIA	Bairro 3 Macia	Domestic worker	Basic secondary level	21	1	2nd trimester	20	≥4	2nd gestational trimester	Adherent	1	Normal
CENTRO DE SAUDE DA MACIA	Bairro 1 Macia	Domestic worker	primary level	20	1	3rd trimester	34	1	3rd gestational trimester	Adherent	1	Normal
CENTRO DE SAUDE DA MACIA	Bairro 2 Macia	public servant	Medium secondary level	32	1	2nd trimester	24	≥4	2nd gestational trimester	Adherent	2	Normal
CENTRO DE SAUDE DA MACIA	Bairro 5 Macia	public servant	primary level	34	2	2nd trimester	17	≥4	2nd gestational trimester	Defaulter	2	Normal
CENTRO DE SAUDE DA MACIA	Bairro 5 Macia	Domestic worker	No formal education	32	≥4	2nd trimester	20	≥4	2nd gestational trimester	Adherent	2	Normal
CENTRO DE SAUDE DA MACIA	Bairro 4 Macia	Domestic worker	Medium secondary level	24	1	2nd trimester	20	≥4	2nd gestational trimester	Adherent	2	Normal
CENTRO DE SAUDE DA MACIA	Bairro 2 Macia	Self employed	Basic secondary level	25	2	Without prenatal consultation	Sem Consulta Pre-Natal	Nenhuma	Without ART	Lost to follow up	1	N/A
CENTRO DE SAUDE DA MACIA	Bairro 2 Macia	Domestic worker	No formal education	29	1	2nd trimester	24	3	2nd gestational trimester	Adherent	2	Normal
CENTRO DE SAUDE DA MACIA	Bairro 1 Macia	Domestic worker	primary level	28	3	2nd trimester	20	≥4	2nd gestational trimester	Adherent	2	Normal
CENTRO DE SAUDE DA MACIA	Bairro 1 Macia	Domestic worker	primary level	29	2	2nd trimester	20	≥4	2nd gestational trimester	Adherent	3	Normal
CENTRO DE SAUDE DA MACIA	Bairro 6 Macia	Student	Medium secondary level	24	2	Without prenatal consultation	Sem Consulta Pre-Natal	Nenhuma	Post partum	Without ART during gestation	2	N/A
CENTRO DE SAUDE DA MACIA	Bairro 1 Macia	Domestic worker	Basic secondary level	22	1	2nd trimester	22	3	2nd gestational trimester	Defaulter	1	Normal
CENTRO DE SAUDE DA MACIA	Bairro 5 Macia	Domestic worker	primary level	29	≥4	Without prenatal consultation	Sem Consulta Pre-Natal	Nenhuma	Post partum	Lost to follow up	1	N/A
CENTRO DE SAUDE DA MACIA	Bairro 4 Macia	public servant	Medium secondary level	27	2	2nd trimester	15	≥4	2nd gestational trimester	Adherent	1	Normal
CENTRO DE SAUDE DA MACIA	Bairro 3 Macia	Domestic worker	primary level	22	1	3rd trimester	28	3	3rd gestational trimester	Lost to follow up	1	Normal
CENTRO DE SAUDE DA MACIA	Bairro 4 Macia	Domestic worker	primary level	26	2	2nd trimester	19	≥4	2nd gestational trimester	Adherent	2	Normal
CENTRO DE SAUDE DA MACIA	Bairro 5 Macia	Self employed	primary level	34	≥4	Without prenatal consultation	Sem Consulta Pre-Natal	Nenhuma	Post partum	Without ART during gestation	1	N/A
CENTRO DE SAUDE DA MACIA	Bairro 4 Macia	Domestic worker	primary level	20	1	Without prenatal consultation	Sem Consulta Pre-Natal	Nenhuma	Post partum	Without ART during gestation	1	N/A
CENTRO DE SAUDE DA MACIA	Bairro 2 Macia	Domestic worker	primary level	23	1	3rd trimester	41	1	Before gestation	Lost to follow up	1	No information
CENTRO DE SAUDE DA MACIA	Bairro 4 Macia	Domestic worker	Medium secondary level	25	1	2nd trimester	19	≥4	2nd gestational trimester	Adherent	1	Normal
CENTRO DE SAUDE DA MACIA	Bairro 1 Macia	Domestic worker	Basic secondary level	24	1	2nd trimester	21	≥4	2nd gestational trimester	Adherent	1	Normal
CENTRO DE SAUDE DA MACIA	Bairro 6 Macia	Domestic worker	primary level	35	3	2nd trimester	21	≥4	2nd gestational trimester	Adherent	1	Normal
CENTRO DE SAUDE DA MACIA	Bairro 3 Macia	Domestic worker	No formal education	32	1	2nd trimester	20	≥4	2nd gestational trimester	Adherent	1	Normal
CENTRO DE SAUDE DA MACIA	Bairro 4 Macia	Domestic worker	primary level	20	≥4	3rd trimester	34	3	3rd gestational trimester	Adherent	2	Normal
CENTRO DE SAUDE DA MACIA	Bairro 3 Macia	Domestic worker	No formal education	31	≥4	3rd trimester	25	≥4	3rd gestational trimester	Adherent	2	Normal
CENTRO DE SAUDE DA MACIA	Bairro 4 Macia	Domestic worker	primary level	24	3	2nd trimester	16	≥4	2nd gestational trimester	Adherent	2	Normal
CENTRO DE SAUDE DA MACIA	Bairro 1 Macia	Domestic worker	primary level	36	≥4	2nd trimester	18	≥4	2nd gestational trimester	Adherent	3	Normal
CENTRO DE SAUDE DA MACIA	Bairro 1 Macia	Domestic worker	Basic secondary level	22	1	3rd trimester	28	3	3rd gestational trimester	Adherent	2	Normal
CENTRO DE SAUDE DA MACIA	Bairro 1 Macia	Domestic worker	primary level	40	3	3rd trimester	30	2	Before gestation	Adherent	3	Normal
CENTRO DE SAUDE DA MACIA	Bairro 1 Macia	Domestic worker	primary level	36	1	3rd trimester	28	3	3rd gestational trimester	Adherent	1	Normal
CENTRO DE SAUDE DA MACIA	Bairro 4 Macia	Domestic worker	primary level	26	2	2nd trimester	14	≥4	2nd gestational trimester	Adherent	2	Normal
CENTRO DE SAUDE DA MACIA	Bairro 3 Macia	Domestic worker	primary level	23	2	2nd trimester	24	3	2nd gestational trimester	Adherent	3	Normal
CENTRO DE SAUDE DA MACIA	MESSANO	Domestic worker	Basic secondary level	22	1	3rd trimester	37	2	3rd gestational trimester	Adherent	2	Normal
CENTRO DE SAUDE DA MACIA	Magul	Domestic worker	primary level	26	2	2nd trimester	20	≥4	2nd gestational trimester	Adherent	2	Normal
CENTRO DE SAUDE DA MACIA	Bairro 6 Macia	Domestic worker	Basic secondary level	23	2	2nd trimester	14	≥4	2nd gestational trimester	Adherent	1	Normal
CENTRO DE SAUDE DA MACIA	Bairro 4 Macia	Domestic worker	No formal education	21	3	2nd trimester	22	3	2nd gestational trimester	Adherent	2	Normal
CENTRO DE SAUDE DA MACIA	Bairro 1 Macia	Domestic worker	Basic secondary level	30	3	2nd trimester	20	≥4	Before gestation	Adherent	1	Normal
CENTRO DE SAUDE DA MACIA	Bairro 1 Macia	Student	Basic secondary level	19	1	2nd trimester	24	≥4	2nd gestational trimester	Adherent	1	Normal
CENTRO DE SAUDE DA MACIA	Bairro 4 Macia	Domestic worker	primary level	23	2	3rd trimester	35	2	3rd gestational trimester	Adherent	1	Normal
CENTRO DE SAUDE DA MACIA	Bairro 3 Macia	Self employed	primary level	30	2	2nd trimester	20	3	2nd gestational trimester	Adherent	2	Normal
CENTRO DE SAUDE DA MACIA	Bairro 5 Macia	Domestic worker	primary level	34	3	2nd trimester	20	≥4	2nd gestational trimester	Adherent	1	Normal
CENTRO DE SAUDE DA MACIA	Bairro 1 Macia	Student	Basic secondary level	22	1	2nd trimester	24	3	2nd gestational trimester	Adherent	1	Normal
CENTRO DE SAUDE DA MACIA	Bairro 1 Macia	Domestic worker	No formal education	23	1	2nd trimester	13	≥4	2nd gestational trimester	Adherent	2	Normal
CENTRO DE SAUDE DA MACIA	MESSANO	Domestic worker	primary level	28	2	2nd trimester	18	≥4	2nd gestational trimester	Adherent	1	Normal
CENTRO DE SAUDE DA MACIA	Uampaco	Domestic worker	No formal education	33	≥4	2nd trimester	20	≥4	2nd gestational trimester	Adherent	3	Normal
CENTRO DE SAUDE DA MACIA	Bairro 2 Macia	Domestic worker	Basic secondary level	32	≥4	Without prenatal consultation	Sem Consulta Pre-Natal	Nenhuma	Post partum	Without ART during gestation	4	Moderate Acute Malnutrition
CENTRO DE SAUDE DA MACIA	Bairro 5 Macia	Domestic worker	primary level	33	3	2nd trimester	24	3	2nd gestational trimester	Defaulter	2	Normal
CENTRO DE SAUDE DA MACIA	Bairro 5 Macia	Self employed	primary level	38	≥4	3rd trimester	32	2	Before gestation	Lost to follow up	1	Normal
CENTRO DE SAUDE DA MACIA	Bairro 4 Macia	Domestic worker	primary level	36	≥4	3rd trimester	34	2	Before gestation	Lost to follow up	2	Normal
CENTRO DE SAUDE DA MACIA	Bairro 1 Macia	Domestic worker	primary level	34	≥4	2nd trimester	24	≥4	Before gestation	Adherent	1	Normal
CENTRO DE SAUDE DA MACIA	Bairro 1 Macia	Domestic worker	primary level	26	3	2nd trimester	22	≥4	Before gestation	Adherent	2	Normal
CENTRO DE SAUDE DA MACIA	Tchacula	Domestic worker	primary level	23	2	2nd trimester	24	3	2nd gestational trimester	Defaulter	3	Normal
CENTRO DE SAUDE DA MACIA	Bairro 2 Macia	Self employed	Basic secondary level	26	3	3rd trimester	28	3	3rd gestational trimester	Defaulter	2	Normal
CENTRO DE SAUDE DA MACIA	Uampaco	Domestic worker	No formal education	45	2	2nd trimester	16	≥4	2nd gestational trimester	Adherent	3	Normal
CENTRO DE SAUDE DA MACIA	Bairro 2 Macia	Domestic worker	primary level	29	≥4	3rd trimester	26	≥4	Before gestation	Adherent	1	Normal
CENTRO DE SAUDE DA MACIA	Bairro 4 Macia	Domestic worker	Basic secondary level	22	2	2nd trimester	20	≥4	Before gestation	Adherent	1	Normal
CENTRO DE SAUDE DA MACIA	Menguelene	Domestic worker	Basic secondary level	29	3	2nd trimester	24	≥4	2nd gestational trimester	Adherent	1	Normal

Genital infections during gestation	Tuberculosis history during gestation/breastfeeding	Viral load during gestation/breastfe	CD4 during gestation/breastfeeding	Age of neonate at birth	Type of delivery	where did the delivery take plac	Sex	birth weight	ART prophylaxis	type of nutrition	Age stopped breastfeeding (m
No	No	≥ 1000 cp	≥ 200 cells	Term	natural birth	Maternity/hospital	Female	≥ 2500 gr	Yes	Exclusive breastfeeding	N/A
No	No	≥ 1000 cp	≥ 200 cells	Term	natural birth	Maternity/hospital	Female	≥ 2500 gr	Yes	Exclusive breastfeeding	N/A
No	No	≥ 1000 cp	≥ 200 cells	Term	natural birth	Maternity/hospital	Female	≥ 2500 gr	Yes	Exclusive breastfeeding	N/A
No	No	≥ 1000 cp	< 200 cells	Term	natural birth	Maternity/hospital	Male	≥ 2500 gr	Yes	Exclusive breastfeeding	N/A
No	No	Not done	< 200 cells	Term	natural birth	Maternity/hospital	Female	≥ 2500 gr	Yes	Exclusive breastfeeding	N/A
No	No	< 1000 cp	≥ 200 cells	Term	natural birth	outside of hospital	Female	≥ 2500 gr	Yes	Exclusive breastfeeding	9
No	No	< 1000 cp	≥ 200 cells	Term	natural birth	Maternity/hospital	Female	≥ 2500 gr	Yes	Exclusive breastfeeding	12
No	No	< 1000 cp	≥ 200 cells	Term	natural birth	Maternity/hospital	Male	≥ 2500 gr	Yes	mixed feeding	12
No	No	< 1000 cp	≥ 200 cells	Term	natural birth	Maternity/hospital	Female	≥ 2500 gr	Yes	Exclusive breastfeeding	12
No	No	< 1000 cp	≥ 200 cells	Term	natural birth	Maternity/hospital	Female	≥ 2500 gr	Yes	Exclusive breastfeeding	12
No	No	< 1000 cp	≥ 200 cells	Term	natural birth	Maternity/hospital	Female	≥ 2500 gr	Yes	Exclusive breastfeeding	9
No	No	< 1000 cp	≥ 200 cells	Term	natural birth	Maternity/hospital	Male	≥ 2500 gr	Yes	Exclusive breastfeeding	12
No	No	≥ 1000 cp	≥ 200 cells	Term	natural birth	Maternity/hospital	Male	≥ 2500 gr	Yes	Exclusive breastfeeding	10
No	No	< 1000 cp	≥ 200 cells	Term	natural birth	Maternity/hospital	Male	≥ 2500 gr	Yes	Exclusive breastfeeding	10
No	No	< 1000 cp	≥ 200 cells	Term	natural birth	Maternity/hospital	Female	≥ 2500 gr	Yes	Exclusive breastfeeding	12
No	No	< 1000 cp	≥ 200 cells	Term	natural birth	Maternity/hospital	Male	≥ 2500 gr	Yes	Exclusive breastfeeding	10
No	No	< 1000 cp	≥ 200 cells	Term	natural birth	Maternity/hospital	Male	≥ 2500 gr	Yes	Exclusive breastfeeding	9
No	No	< 1000 cp	≥ 200 cells	Pre term	C-section	Maternity/hospital	Female	≥ 2500 gr	Yes	Exclusive breastfeeding	10
No	No	< 1000 cp	< 200 cells	Term	natural birth	Maternity/hospital	Female	≥ 2500 gr	Yes	Artificial feeding	N/A
No	No	< 1000 cp	≥ 200 cells	Term	natural birth	Maternity/hospital	Female	≥ 2500 gr	Yes	Exclusive breastfeeding	9
No	No	< 1000 cp	≥ 200 cells	post term	natural birth	Maternity/hospital	Male	≥ 2500 gr	Yes	Exclusive breastfeeding	12
No	No	< 1000 cp	≥ 200 cells	Term	natural birth	Maternity/hospital	Female	≥ 2500 gr	Yes	Exclusive breastfeeding	9
No	No	< 1000 cp	≥ 200 cells	Term	natural birth	Maternity/hospital	Male	≥ 2500 gr	Yes	Exclusive breastfeeding	10
No	No	< 1000 cp	≥ 200 cells	Term	natural birth	Maternity/hospital	Male	≥ 2500 gr	Yes	Exclusive breastfeeding	9
No	No	< 1000 cp	≥ 200 cells	Term	natural birth	Maternity/hospital	Female	≥ 2500 gr	Yes	Exclusive breastfeeding	10
No	No	< 1000 cp	≥ 200 cells	Term	natural birth	Maternity/hospital	Male	≥ 2500 gr	Yes	Exclusive breastfeeding	10
No	No	≥ 1000 cp	≥ 200 cells	Pre term	natural birth	Maternity/hospital	Female	< 2500 gr	Yes	Exclusive breastfeeding	12
No	No	< 1000 cp	≥ 200 cells	Term	natural birth	Maternity/hospital	Female	≥ 2500 gr	Yes	Exclusive breastfeeding	9
Yes	No	< 1000 cp	≥ 200 cells	Term	natural birth	Maternity/hospital	Female	≥ 2500 gr	Yes	Exclusive breastfeeding	9
No	No	< 1000 cp	≥ 200 cells	Term	natural birth	Maternity/hospital	Male	≥ 2500 gr	Yes	Exclusive breastfeeding	10
No	No	< 1000 cp	< 200 cells	Term	natural birth	Maternity/hospital	Male	≥ 2500 gr	Yes	Exclusive breastfeeding	10
No	No	< 1000 cp	≥ 200 cells	Term	natural birth	Maternity/hospital	Male	≥ 2500 gr	Yes	Exclusive breastfeeding	11
No	No	< 1000 cp	≥ 200 cells	Term	natural birth	Maternity/hospital	Male	≥ 2500 gr	Yes	Exclusive breastfeeding	10
No	No	< 1000 cp	≥ 200 cells	Term	natural birth	Maternity/hospital	Female	≥ 2500 gr	Yes	Artificial feeding	No information
No	No	Not done	≥ 200 cells	Pre term	natural birth	Maternity/hospital	Female	No information	Yes	Exclusive breastfeeding	N/A
No	No	< 1000 cp	≥ 200 cells	Term	natural birth	Maternity/hospital	Female	≥ 2500 gr	Yes	Exclusive breastfeeding	7
No	No	< 1000 cp	≥ 200 cells	Term	natural birth	Maternity/hospital	Male	≥ 2500 gr	Yes	Exclusive breastfeeding	9
No	No	< 1000 cp	≥ 200 cells	Term	natural birth	Maternity/hospital	Female	≥ 2500 gr	Yes	Artificial feeding	N/A
No	No	< 1000 cp	≥ 200 cells	Term	natural birth	Maternity/hospital	Female	≥ 2500 gr	Yes	Exclusive breastfeeding	7
N/A	N/A	Not done	≥ 200 cells	Term	natural birth	Maternity/hospital	Female	≥ 2500 gr	Yes	Exclusive breastfeeding	N/A
No	No	≥ 1000 cp	< 200 cells	Term	natural birth	Maternity/hospital	Female	≥ 2500 gr	Yes	Exclusive breastfeeding	N/A
No	No	< 1000 cp	< 200 cells	Term	natural birth	outside of hospital	Male	≥ 2500 gr	Yes	Exclusive breastfeeding	9
No	No	< 1000 cp	< 200 cells	Term	natural birth	Maternity/hospital	Female	≥ 2500 gr	Yes	Exclusive breastfeeding	9
N/A	N/A	N/A	N/A	Term	natural birth	Maternity/hospital	Female	≥ 2500 gr	Yes	mixed feeding	N/A
No	No	Not done	≥ 200 cells	Term	natural birth	Maternity/hospital	Male	≥ 2500 gr	Yes	Exclusive breastfeeding	N/A
N/A	N/A	Not done	≥ 200 cells	Pre term	natural birth	Maternity/hospital	Female	≥ 2500 gr	Yes	Exclusive breastfeeding	N/A
No	No	≥ 1000 cp	≥ 200 cells	Term	natural birth	Maternity/hospital	Female	≥ 2500 gr	Yes	Exclusive breastfeeding	9
No	No	Not done	≥ 200 cells	Term	natural birth	Maternity/hospital	Female	≥ 2500 gr	Yes	mixed feeding	No information
No	No	≥ 1000 cp	≥ 200 cells	Pre term	natural birth	Maternity/hospital	Female	≥ 2500 gr	Yes	Exclusive breastfeeding	N/A
N/A	N/A	N/A	N/A	No information	natural birth	Maternity/hospital	Female	No information	No	mixed feeding	N/A
N/A	N/A	N/A	N/A	Term	natural birth	Maternity/hospital	Male	≥ 2500 gr	Yes	Exclusive breastfeeding	N/A
No	No	≥ 1000 cp	≥ 200 cells	Term	natural birth	Maternity/hospital	Male	≥ 2500 gr	Yes	Exclusive breastfeeding	9
No	No	≥ 1000 cp	< 200 cells	post term	natural birth	Maternity/hospital	Female	≥ 2500 gr	Yes	Exclusive breastfeeding	N/A
No	No	≥ 1000 cp	≥ 200 cells	Term	natural birth	Maternity/hospital	Female	≥ 2500 gr	Yes	Exclusive breastfeeding	8
No	No	≥ 1000 cp	≥ 200 cells	Term	natural birth	Maternity/hospital	Male	≥ 2500 gr	Yes	Exclusive breastfeeding	No information
No	No	≥ 1000 cp	≥ 200 cells	Term	natural birth	Maternity/hospital	Female	≥ 2500 gr	Yes	Exclusive breastfeeding	No information
No	No	< 1000 cp	≥ 200 cells	Term	natural birth	Maternity/hospital	Female	< 2500 gr	Yes	Exclusive breastfeeding	N/A
No	No	≥ 1000 cp	≥ 200 cells	Term	natural birth	outside of hospital	Female	≥ 2500 gr	Yes	Exclusive breastfeeding	N/A
No	No	< 1000 cp	≥ 200 cells	Term	natural birth	Maternity/hospital	Female	≥ 2500 gr	Yes	Exclusive breastfeeding	9
No	No	< 1000 cp	≥ 200 cells	Term	natural birth	Maternity/hospital	Male	≥ 2500 gr	Yes	Exclusive breastfeeding	9
No	No	< 1000 cp	≥ 200 cells	Term	natural birth	Maternity/hospital	Male	≥ 2500 gr	Yes	Exclusive breastfeeding	9
No	No	≥ 1000 cp	≥ 200 cells	Term	natural birth	Maternity/hospital	Female	≥ 2500 gr	Yes	Artificial feeding	N/A
No	No	< 1000 cp	≥ 200 cells	Term	natural birth	Maternity/hospital	Male	≥ 2500 gr	Yes	Exclusive breastfeeding	8
No	No	< 1000 cp	≥ 200 cells	Term	natural birth	Maternity/hospital	Male	≥ 2500 gr	Yes	Exclusive breastfeeding	9
No	No	< 1000 cp	≥ 200 cells	Term	natural birth	Maternity/hospital	Male	≥ 2500 gr	Yes	Exclusive breastfeeding	9
No	No	< 1000 cp	≥ 200 cells	Term	natural birth	Maternity/hospital	Male	≥ 2500 gr	Yes	Exclusive breastfeeding	9
No	No	< 1000 cp	≥ 200 cells	Term	natural birth	Maternity/hospital	Female	≥ 2500 gr	Yes	Exclusive breastfeeding	9
No	No	< 1000 cp	≥ 200 cells	Term	natural birth	Maternity/hospital	Female	≥ 2500 gr	Yes	Exclusive breastfeeding	9
No	No	< 1000 cp	≥ 200 cells	Term	natural birth	Maternity/hospital	Male	≥ 2500 gr	Yes	Exclusive breastfeeding	9
No	No	< 1000 cp	≥ 200 cells	Term	natural birth	Maternity/hospital	Female	≥ 2500 gr	Yes	Exclusive breastfeeding	9
No	No	< 1000 cp	≥ 200 cells	Term	natural birth	Maternity/hospital	Female	≥ 2500 gr	Yes	Exclusive breastfeeding	10
No	No	< 1000 cp	≥ 200 cells	Term	natural birth	Maternity/hospital	Female	≥ 2500 gr	Yes	Exclusive breastfeeding	9
No	No	< 1000 cp	< 200 cells	Term	natural birth	Maternity/hospital	Male	< 2500 gr	Yes	Exclusive breastfeeding	9
No	No	< 1000 cp	≥ 200 cells	Term	natural birth	Maternity/hospital	Male	≥ 2500 gr	Yes	Exclusive breastfeeding	7
No	No	< 1000 cp	≥ 200 cells	Term	natural birth	Maternity/hospital	Female	< 2500 gr	Yes	Exclusive breastfeeding	9
No	No	< 1000 cp	≥ 200 cells	Term	natural birth	Maternity/hospital	Female	≥ 2500 gr	Yes	Exclusive breastfeeding	9
No	No	< 1000 cp	≥ 200 cells	Term	natural birth	Maternity/hospital	Male	≥ 2500 gr	Yes	Exclusive breastfeeding	9
No	No	< 1000 cp	≥ 200 cells	Term	natural birth	Maternity/hospital	Male	≥ 2500 gr	Yes	Exclusive breastfeeding	10
No	No	Not done	< 200 cells	Term	natural birth	outside of hospital	Female	≥ 2500 gr	Yes	Exclusive breastfeeding	N/A
No	No	≥ 1000 cp	≥ 200 cells	Term	natural birth	Maternity/hospital	Female	≥ 2500 gr	Yes	Exclusive breastfeeding	No information
No	No	≥ 1000 cp	≥ 200 cells	Pre term	natural birth	Maternity/hospital	Female	≥ 2500 gr	Yes	Exclusive breastfeeding	N/A
No	No	≥ 1000 cp	< 200 cells	Term	natural birth	Maternity/hospital	Female	< 2500 gr	Yes	Exclusive breastfeeding	N/A
No	No	< 1000 cp	≥ 200 cells	post term	natural birth	Maternity/hospital	Female	≥ 2500 gr	Yes	Exclusive breastfeeding	9
No	No	< 1000 cp	≥ 200 cells	Term	natural birth	Maternity/hospital	Male	≥ 2500 gr	Yes	Exclusive breastfeeding	9
No	No	≥ 1000 cp	≥ 200 cells	Term	natural birth	Maternity/hospital	Female	≥ 2500 gr	Yes	mixed feeding	9
No	No	≥ 1000 cp	≥ 200 cells	Term	natural birth	Maternity/hospital	Female	≥ 2500 gr	Yes	Exclusive breastfeeding	10
No	No	< 1000 cp	< 200 cells	Term	natural birth	Maternity/hospital	Female	≥ 2500 gr	Yes	Exclusive breastfeeding	8
No	No	< 1000 cp	≥ 200 cells	Term	natural birth	Maternity/hospital	Male	≥ 2500 gr	Yes	Exclusive breastfeeding	10
No	No	< 1000 cp	≥ 200 cells	Term	natural birth	Maternity/hospital	Female	≥ 2500 gr	Yes	Exclusive breastfeeding	8
No	No	< 1000 cp	≥ 200 cells	Term	natural birth	Maternity/hospital	Male	≥ 2500 gr	Yes	Exclusive breastfeeding	10

nutritional status	Final results of HIV status	Age at diagnosis (Months)	observations
Normal	Positive	1	
Normal	Positive	2	
Normal	Positive	1	ART lost to follow up during breastfeeding period
Normal	Positive	1	
Normal	Positive	1	
Normal	Negative	13	
Normal	Negative	14	
Normal	Negative	17	
Normal	Negative	19	
Normal	Negative	15	
Normal	Negative	12	
Normal	Negative	14	
Normal	Negative	12	
Normal	Negative	18	
Normal	Negative	14	
Normal	Negative	12	
Normal	Negative	12	
Normal	Negative	12	
Normal	Negative	18	
Normal	Negative	11	
Normal	Negative	14	
Normal	Negative	12	
Mild Acute Malnutrition	Positive	13	
Normal	Negative	12	
Mild Acute Malnutrition	Negative	12	
Normal	Negative	17	
Normal	Negative	14	
Normal	Negative	12	
Normal	Negative	11	
Normal	Negative	12	
Mild Acute Malnutrition	Negative	12	
Normal	Negative	13	
Normal	Negative	12	
Normal	Negative	1	
Normal	Positive	1	
Normal	Negative	9	
Normal	Negative	12	
Normal	Negative	9	
Normal	Negative	11	
Normal	Positive	12	Mother with no PMTCT
Normal	Positive	1	
Normal	Negative	11	
Normal	Negative	12	
Normal	Positive	12	Mother with no PMTCT
Severe Acute Malnutrition	Positive	2	
Normal	Positive	12	
Normal	Positive	12	
Normal	Positive	15	
Normal	Positive	1	
N/A	Positive	12	Mother with no PMTCT
N/A	Positive	10	Mother with no PMTCT
Normal	Positive	9	Child born 1 day after ANC card opening, 1st PCR negative
Normal	Positive	1	
Normal	Positive	9	1st PCR negative
Normal	Positive	9	1st PCR negative
Normal	Positive	5	
Normal	Positive	1	
Normal	Positive	1	
Normal	Negative	11	
Normal	Negative	13	
Normal	Negative	12	
Mild Acute Malnutrition	Negative	10	
Normal	Negative	10	
Normal	Negative	12	
Normal	Negative	12	
Normal	Negative	14	
Normal	Negative	11	
Normal	Negative	12	
Normal	Negative	13	
Normal	Negative	13	
Normal	Negative	12	
Normal	Negative	14	
Normal	Negative	11	
Normal	Negative	9	
Normal	Negative	13	
Normal	Negative	13	
Normal	Negative	11	
Normal	Negative	12	
Normal	Positive	1	Mother died after 1st month post partum and ART initiation
Normal	Positive	5	
Normal	Positive	1	
Normal	Positive	1	
Normal	Negative	13	
Normal	Negative	12	
Normal	Positive	12	1st PCR negative
Normal	Positive	12	1st PCR negative
Normal	Negative	12	
Normal	Negative	13	
Normal	Negative	13	
Normal	Negative	12	
